# Electrospun Fiber Pads of Cellulose Acetate and Essential Oils with Antimicrobial Activity

**DOI:** 10.3390/nano7040084

**Published:** 2017-04-12

**Authors:** Ioannis L. Liakos, Alina Maria Holban, Riccardo Carzino, Simone Lauciello, Alexandru Mihai Grumezescu

**Affiliations:** 1 Smart Materials Group, Nanophysics Department, Istituto Italiano di Tecnologia (IIT), Via Morego 30, 16163 Genoa, Italy; riccardo.carzino@iit.it; 2 Center for Micro-BioRobotics, Istituto Italiano di Tecnologia (IIT), Viale Rinaldo Piaggio 34, 56025 Pontedera, Pisa, Italy; 3 Department of Microbiology and Immunology, Faculty of Biology, University of Bucharest, Aleea Portocalelor, No. 1-3, 060101 Bucharest, Romania; alina_m_h@yahoo.com; 4 Department of Science and Engineering of Oxide Materials and Nanomaterials, Faculty of Applied Chemistry and Materials Science, University Politehnica of Bucharest, Polizu Street No. 1-7, 011061 Bucharest, Romania; grumezescu@yahoo.com; 5 Nanochemistry Department, Istituto Italiano di Tecnologia (IIT), Via Morego 30, 16163 Genoa, Italy; simone.lauciello@iit.it

**Keywords:** cellulose acetate, electrospun fibers, essential oils, antibacterial nanofibers, wound dressings

## Abstract

The method of electrospinning was used to create nanofibers made of cellulose acetate (CA) and essential oils (EOs). CA polymer at 15% *w*/*v* was dissolved in acetone and then 1% or 5% *v*/*v* of EOs was added to the polymer solution. The utilized essential oils were rosemary and oregano oils. Then, the CA/EOs in acetone solution were electrospun, creating micro/nanofibers, approximately 700–1500 nm in diameter. Raman spectroscopy was used to detect the attachment of the EOs in the CA electrospun fibers (ESFs). Scanning electron microscopy was used to study the morphology, topography and dimensions of the ESFs. The formed CA/EOs ESFs are found to have good antimicrobial properties against three common microbial species, frequently found in difficult to treat infections: Bacteria species *Staphylococcus aureus*, Escherichia coli and the yeast *Candida albicans*. ESFs with 5% *v*/*v* oregano oil with respect to the initial solution, showed the best antimicrobial and anti-biofilm effects due to the potency of this EO against bacteria and fungi, especially for *Escherichia coli* and *Candida albicans*. This work describes an effective and simple method to prepare CA/EOs ESFs and opens up many new applications of micro/nanofibers such as improved antimicrobial wound dressings, anti-biofilm surfaces, sensors and packaging alternatives.

## 1. Introduction

Antimicrobial resistance is a growing problem, since microorganisms tend to develop mechanisms of adaptation to currently used antimicrobial agents [1]. In the last years, few novel antibiotics have been developed and their development is a difficult process, therefore we are limited to using the available antimicrobial drugs, while bacteria find various ways to resist them [2]. The use of nanotechnology can help to increase the efficacy of the current antimicrobial agents, due to the high surface-to-volume ratio that the nanomaterials can provide [3]. Nano- and micro-fibers can be developed by the electrospinning technique and create materials that can have applications in the biomedical field, such as wound dressings [4] and water filtration in order to remove pathogenic microorganisms [5].

In this work, a natural derived polymer, cellulose acetate (CA), together with some antimicrobial essential oils have been used to create free-standing nanofibers using the electrospinning technique. Cellulose acetate is the acetate ester of cellulose, which is an important structural component of the cell wall of green plants. CA can be easily electrospun, producing ultrafine fibers of sub-micrometer scale. Such CA electrospun fibers (ESFs) have good thermal stability, chemical resistance, biocompatibility and biodegradability; they can therefore be used as wound dressings and can clean water from bacteria (filtering). Other applications of CA ESFs include tissue engineering, drug delivery, separation membrane, sensors and others [6].

In the last decades, a trend was observed for using substances derived from plants and animals as antimicrobial agents [7,8,9]. For example, chlorinated keratin moieties with cellulose acetate electrospun nanofibers have shown very good antimicrobial activity against *S. aureus* and *E. coli* [10]. Keratin/poly(ethylene oxide) electrospun nanofibers have also been loaded with irgasan and demonstrated very good antibacterial activity against *S. aureus* and *E. coli* with moderate irgasan loads (5–7 wt %), especially after 24 h of incubation [11]. Plant-derived antimicrobials such as essential oils (EOs) are produced from steam distillation of plants and have many biomedical properties. One of the properties of some EOs is their antibacterial or antimicrobial activity [12,13,14,15,16,17,18]. Wound dressings with EOs and plant extracts have shown very good antimicrobial activity and thus are very promising candidates against pathogenic bacteria and microorganisms [9,12,13,14,19,20,21,22]. Polymers such as polylactic acid [23] and chitosan/poly(ethylene oxide) [24] have been used with EOs to create electrospun nanofibers with antimicrobial properties. Previous research has shown that CA ESFs with lemongrass, cinnamon and peppermint EOs showed good antibacterial activity against *E. coli* [18]. Cellulose acetate has many advantages, since it is biodegradable, does not dissolve in water, can retain the EOs for a long time and, due to its cellulose origin, looks and feels like cotton and can be used for wound dressing applications [18,25,26,27].

We tried to extend our previous work [18], by using two new essential oils, namely Rosemary and Oregano Eos, in CA ESFs. These two EOs were used since they possess great antibacterial activity [28]. The new developed CA EOs ESFs were tested against numerous pathologic bacteria species such as *S. aureus*, *E. coli* and *C. albicans* to obtain a better picture of how these ESFs with new EOs can be used against more bacterial strains. Such produced ESFs with antimicrobial activity can be very efficient against pathogenic organisms due to their high surface-to-volume ratio that such micro/nanofibers create, but also due to microorganisms being relatively non-resistant towards EOs. The bacteria are estimated to sense much more exposed area of the antimicrobial agent, than that of a simple 2D or 3D film.

## 2. Experimental Section

### 2.1. Materials

EOs of rosemary (R) and oregano (Or) (100% pure) were purchased from Maitreya-Natura (Carpegna, Italy). Cellulose Acetate (CA) (acetyl content of 39.8%; M_W_ = 30 kDa) and acetone were purchased from Sigma-Aldrich, Milano, Italy.

### 2.2. Preparation of CA/EOs ESFs

Solutions of CA with rosemary or oregano essential oil were prepared by dissolving 15% *w*/*v* CA in acetone and then adding 1% or 5% *v*/*v* of the selected EO. Plastic syringes with stainless steel 23-gauge needles were filled with the CA/EO solutions and connected to a syringe pump (NE-1000, New Era Pump Systems Inc., Farmingdale, NY, USA) that was working at flow rates of 3 or 5 mL h^−1^. The solutions were electrospun by using a high voltage power supply (EH40R2.5, Glassman High Voltage Inc., High Bridge, NJ, USA). The produced fibers were collected on an aluminium foil, placed at a distance of 15 cm from the needle. The following parameters of flow rate and voltage were used for the ES process, ensuring the best conditions to provide fibers free of defects and inhomogeneities: 2 mL h^−1^, −120 kV for either CA pristine solution or CA solutions containing EO. The resulting electrospun mats had a thickness of about 0.2 mm.

### 2.3. Micro-Raman Spectroscopy

The chemical analysis of the CA and CA/EOs fibers was carried out by Raman spectroscopy, using a Horiba Jobin–Yvonm Raman operating with a He–Ne laser source (Horiba Scientific, Kyoto, Japan). The wavelength of the laser radiation was 632 nm and the objective used was a 50× with a slit aperture of about 200 μm.

### 2.4. Scanning Electron Microscopy (SEM)

The morphology and the size of the electrospun fibers were analyzed by scanning electron microscopy (SEM). A JEOL JSM-6490LA microscope (Jeol, Peabody, MA, USA) working in high vacuum mode, with an acceleration voltage of 15 kV, was used. A coating of 10 nm Au/Pd was required to prevent charging effects.

### 2.5. Biofilm Formation

*Staphylococcus aureus* ATCC25923, *Escherichia coli* ATCC25922 and *Candida albicans* ATCC 10231 strains (American Type Cell Collection, Manassas, VA, USA) were utilized to assess antimicrobial effect and biofilm formation in the presence of synthesized CA ESFs. Monospecific biofilm development was analyzed at different times of exposure, using sterile 6-well plates (Nunc). Ultraviolet (UV) sterilized MAPLE coated samples were added in 6-well plates containing 2 mL of nutritive broth inoculated with ~10^6^ CFU/mL of microbial suspensions prepared in sterile saline solution. The samples were incubated at 37 °C for 24 h to allow microbial development, attachment to the prepared materials and subsequent biofilms development. After incubation, the samples were carefully washed with sterile saline buffer to remove any unattached microbial cells and then immersed in 1 mL sterile saline buffer. Biofilm cells were detached by vigorous vortexing and pipetting. The resulting biofilm-detached cell suspensions were further diluted and 10 µL of each serial dilution was plated in triplicate on nutritive agar. After 24 h of incubation at 37 °C, viable count was performed and the CFU/mL values for each sample were obtained and plotted on a graph.

## 3. Results

### 3.1. Micro-Raman Spectroscopy

**Figure 1:**
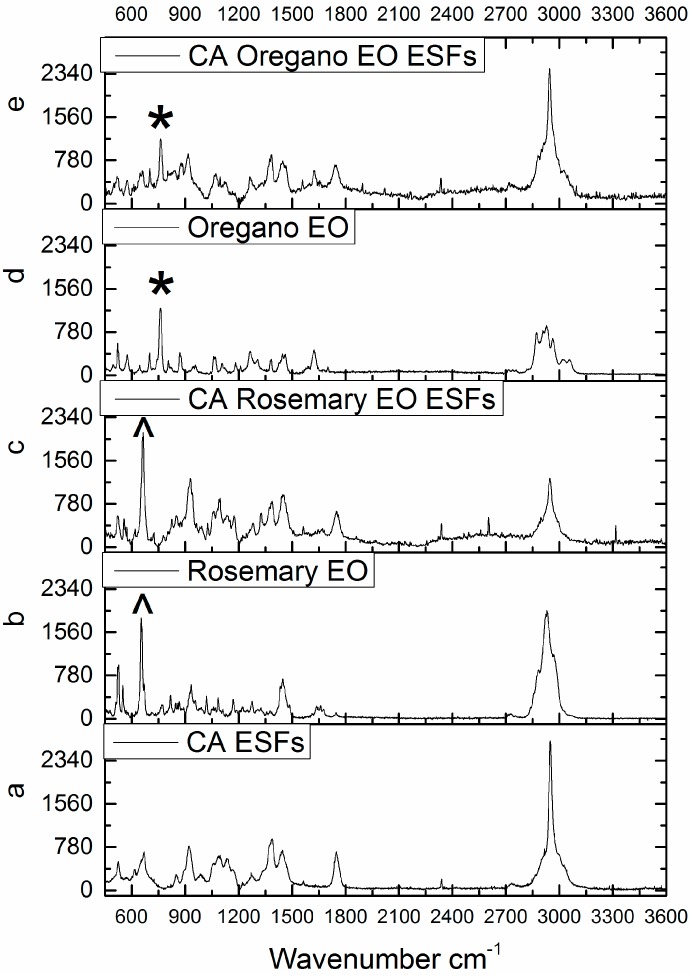
Raman spectroscopy of (**a**) (cellulose acetate) CA (electrospun fibers) ESFs, (**b**) Rosemary (essential oil) EO, (**c**) CA/Rosemary EO ESFs, (**d**) Oregano EO and (**e**) CA/Oregano EO ESFs.

The Raman spectrum of CA ESFs is shown in Figure 1a. The bands at 2950 and 2980 cm^−1^ are assigned to the symmetric and asymmetric CH_2_ vibrations. The characteristic peak that arises from the C=O bonds in acetyl groups of the polymer chains is around 1750 cm^−1^. The peaks at around 1380 and 1450 cm^−1^ arise from the various deformation vibrations of the CA backbone and from bending vibrations of CH_2_ groups in cellulose respectively. The peaks between 1300 and 800 cm^−1^ are mainly assigned to stretching vibrations of C–O groups and asymmetric CC and CO vibrations. The peak around 1122 cm^−1^ is due to COC symmetric ring vibrations [18,29].

The Raman spectra of rosemary EO and CA/Rosemary EO ESFs are shown in Figure 1b,c respectively. Rosemary EO shows a characteristic peak at around 650 cm^−1^; this peak is clearly shown also in CA rosemary ESFs (denoted by the symbol ^). This peak arises from the vibrations of camphor molecules that exist in rosemary EO [30].

The Raman spectra of oregano EO and CA/Oregano EO ESFs are shown in Figure 1d,e respectively. The peak of oregano oil at around 740 cm^−1^ is a distinct peak with high intensity which is also seen clearly in CA Oregano EO ESFs spectra (denoted by the symbol *). This peak arises from ring stretching vibrations of aromatic molecules found in oregano EO [31].

From the above data, it can easily be concluded that the EOs have been well grafted into the CA polymer matrix and they present in the ESFs. Additionally, it is indicated here that the ESFs of CA with EOs have a distinct perfume that originates from the aromatic character of the EOs used.

### 3.2. Scanning Electron Microscopy (SEM)

**Figure 2:**
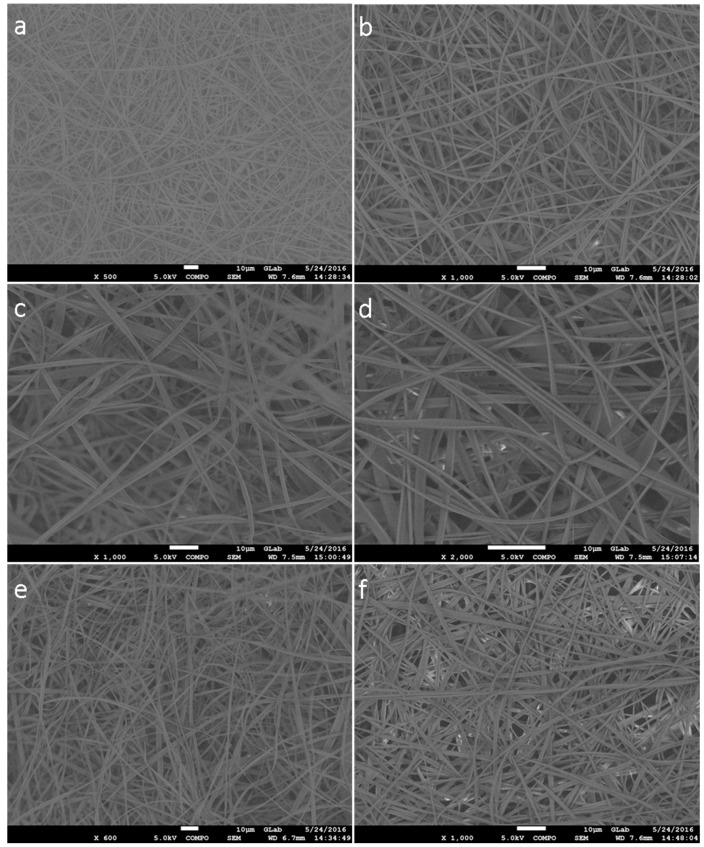
SEM of (**a**,**b**) CA ESFs; (**c**) CA 1% Rosemary EO, (**d**) CA 5% Rosemary EO; (**e**) CA 1% Oregano EO and (**f**) CA 5% Oregano EO.

In Figure 2a,b, the SEM images of representative CA ESFs are shown. As observed, the fibers are uniform and free of defects or beads.

Similarly, the ESFs of CA/Rosemary EO at concentrations 1% and 5% are shown in Figure 2. The fibers are uniform, free of any defect with diameters ranging from 0.7 μm to 1.5 μm approximately. In Figure 2, the CA/Oregano EO ESFs with 1 and 5% concentration of EO are presented, and as can be seen, they are similar to the previous fibers of rosemary.

**Figure 3:**
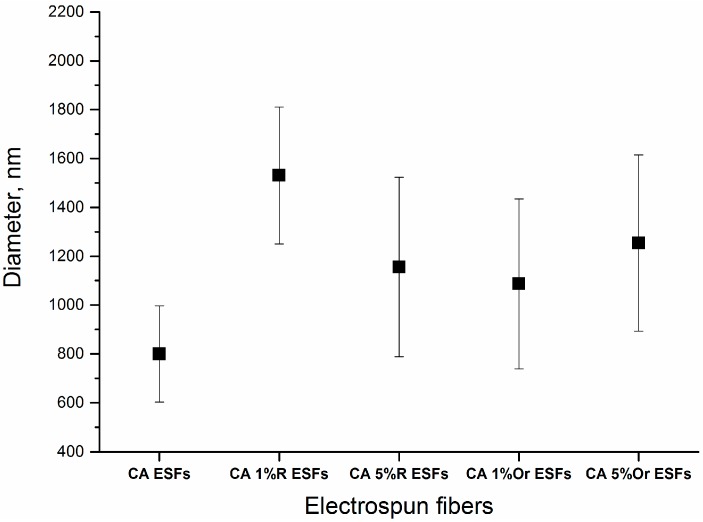
Diameter of ESFs; R = Rosemary, Or = Oregano.

From the above SEM data, it is observed that the addition of EOs did not change the quality or dimensions of CA ESFs. Therefore, the addition of the two EOs, with the aforementioned experimental set-up to make ESFs, produced fibers that resemble each other and resemble the reference (CA ESFs), i.e., were free of any defect and continuous. The diameters of all the fibers ranged from 800 to 1600 nm, as calculated for 10 fibers for each image and illustrated in Figure 3. The fibers loaded with EOs had higher diameters (400–500 nm) than those of bare CA ESFs, due to the EO incorporation into the CA polymer.

### 3.3. Biofilm Formation

**Figure 4:**
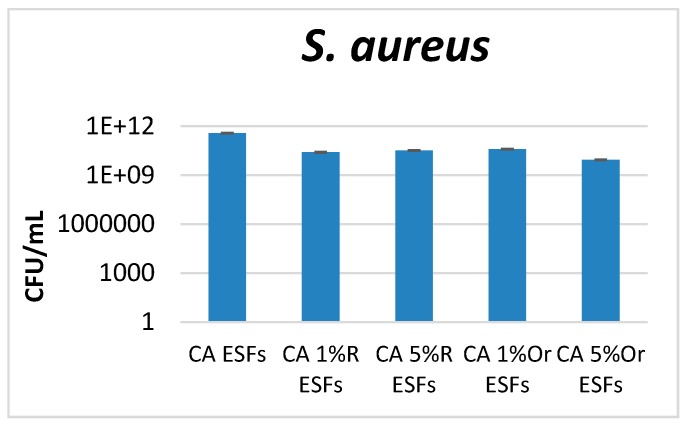
Graphic representation of the CFU/mL values obtained after assessing viable counts of *S. aureus* microbial cells obtained from biofilms developed for 24 h on EO-containing and control CA ESFs; R = Rosemary, Or = Oregano.

**Figure 5:**
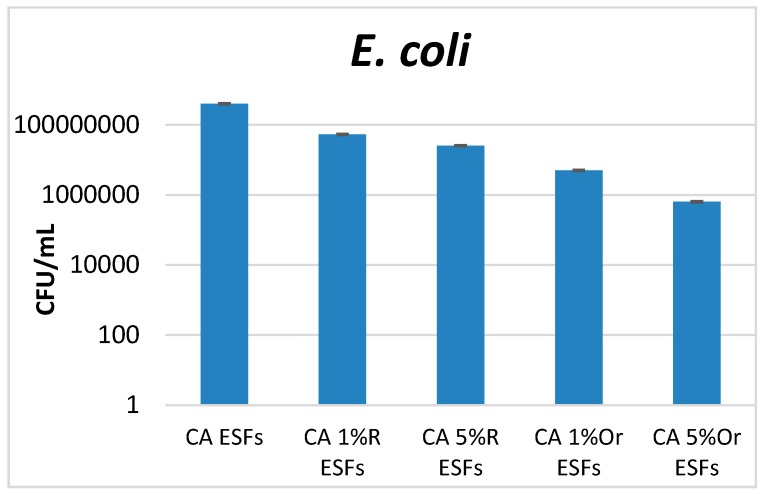
Graphic representation of the CFU/mL values obtained after assessing viable counts of *E. coli* microbial cells obtained from biofilms developed for 24 h on EO-containing and control CA ESFs; R = Rosemary, Or = Oregano.

Antimicrobial tests were performed on three microbial species, two bacteria (one Gram-positive, *S. aureus*, one Gram-negative, *E. coli*) and one yeast (*C. albicans*). These microorganisms are the most investigated model microorganisms for studying pathogenicity, resistance, infectious process development and biofilm formation. Moreover, they are also the most frequent and versatile opportunistic pathogens, causing numerous community and hospital-acquired infections. *S. aureus* is one of the most frequent Gram-positive etiologies in skin and wound infections, with its prevalence being related to the fact that about one in three people carry this bacterium in their nose and pharynx. Moreover, this species causes severe infections in critical care patients, with resistant strains (such as MRSA = methicillin resistant *S. aureus*) often being isolated [32]. The ability of *S. aureus* to produce highly organized multicellular communities (biofilms)—which are very resistant to any treatment with antimicrobial drugs, on various medical surfaces, indwelling devices and open wounds—significantly limits the therapeutic options [33]. *E. coli* is a Gram-negative study model, very useful in the investigation of biofilm formation, causing several types of severe infections, from gastrointestinal diseases to resistant urinary tract infection [34]. *Candida albicans* represents the most frequent yeast etiology infection in hospitalized and immunocompromised patients. Also, this species causes various infections associated with antibiotic therapy and dysbiosis, representing a risk factor for the development of biofilms on implantable devices [35]. Our results demonstrated that all obtained materials containing essential oils inhibit microbial attachment and biofilm formation, the results being influenced by the essential oil and the tested microbial strain. For analyzed bacteria (*S. aureus* and *E. coli*), it was observed that CA Oregano EO ESFs manifested the most significant anti-biofilm effect; the higher the amount of EO, the more intense the effect that was developed. This trend was also observed for Rosemary-containing ESFs, but the anti-biofilm effect was lower in the case of *S. aureus* (Figure 4). In the case of *E. coli*, significant differences were highlighted among the obtained EOs, with materials containing oregano EO displaying the highest anti-biofilm effect (Figure 5).

**Figure 6:**
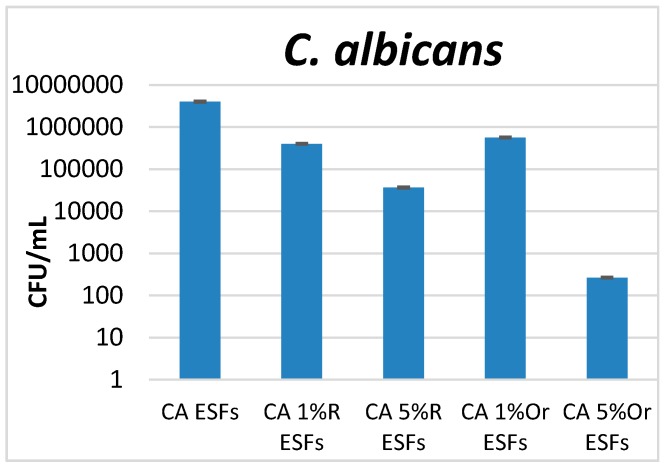
Graphic representation of the CFU/mL values obtained after assessing viable counts of *S. aureus* microbial cells obtained from biofilms developed for 24 h on EO-containing and control CA ESFs; R = Rosemary, Or = Oregano.

While analyzing the development of yeast cells and biofilms, we observed that the most significant inhibitory effect was obtained when an amount of 5% oregano EO was used for the design of ESFs. Moreover, high amounts of rosemary EO have a more intense anti-biofilm effect on *C. albicans* compared with the use of 1% EO, both in the case of rosemary and oregano (Figure 6).

One of the main reasons that oregano oil shows higher antibacterial activity than rosemary oil is that oregano oil consists mainly of carvacrol and thymol [36] which are both substances with high antimicrobial property [37,38]. Rosemary oil consists mainly of alpha-pinene, camphene, eucalyptol and camphre, which seem to have less potency than the substances present in oregano oil. Also, the current data confirm previous research that has shown that rosemary oil is more efficient against Gram-positive bacteria and yeasts (*C. albicans*) than Gram-negative ones (*E. coli*) [39]. On the other hand, oregano oil showed very good antibacterial activity against *C. albicans* and the Gram-negative *E. coli* bacteria strain. It is also noticed that both tested EOs showed antibacterial activity that is reduced as follows (depending on the microbial species) *C. albicans* > *E. coli* > *S. aureus*. *S. aureus* seems to be the most resistant bacteria species to the studied CA/EO ESFs. Other researchers have also pointed out the increasing antimicrobial resistance of *S. aureus* both to antibiotics and antimicrobial plant-derived compounds [32]. The current reported results are in accordance with similar concentrations of EOs when compared with another work of electrospun polyvinyl alcohol/cinnamon EO/β-cyclodextrin [40]. The researchers reported similar antimicrobial activity for *E. coli* but higher antimicrobial activity for *S. aureus* at similar concentrations [40], possibly due to the high potency of cinnamon as an antimicrobial essential oil [14,18]. Similar results to those presented here for *E. coli*, have been obtained with chitosan/cinnamaldehyde/poly(ethylene oxide) nanofibers [24] at similar concentrations to those reported here. When the amount of cinnamaldehyde to the polymers was 1 to 1, the antimicrobial activity against *E. coli* increased significantly [24].

## 4. Conclusions

Cellulose acetate electrospun fibers were successfully formed, but such microfibers lack any biological (i.e., antibacterial) activity. Rosemary and oregano essential oils were efficiently incorporated into the electrospun fibers. Such fibers with the presence of essential oils were uniform, continuous and free of any defects. The antibacterial activity of rosemary and oregano oil was retained in the fibers as the antibacterial tests demonstrated. It seems that with a higher essential oil concentration, the antibacterial activity increases. We demonstrated that the antibacterial activity of electrospun fibers with essential oils is not the same for the three tested bacterial strains. This work showed that oregano EO is more potent against the three studied bacteria compared to rosemary EO, probably due to the high antimicrobial character of the oregano oil molecules, such as carvacol and thymol. The fibers formed with essential oils were effective against all the studied microbial strains with the potency decreasing as follows: *C. albicans* > *E. coli* > *S. aureus*.
